# SIRT1 Protects the Heart from ER Stress-Induced Injury by Promoting eEF2K/eEF2-Dependent Autophagy

**DOI:** 10.3390/cells9020426

**Published:** 2020-02-12

**Authors:** Julie Pires Da Silva, Kevin Monceaux, Arnaud Guilbert, Mélanie Gressette, Jérôme Piquereau, Marta Novotova, Renée Ventura-Clapier, Anne Garnier, Christophe Lemaire

**Affiliations:** 1 Université Paris-Saclay, Inserm, UMR-S 1180, 92296 Châtenay-Malabry, France; 2 Institute of Experimental Endocrinology, Biomedical Centre SAS, 833 06 Bratislava, Slovakia; 3 Université Versailles St-Quentin, Université Paris-Saclay, Inserm, UMR-S 1180, 92296 Châtenay-Malabry, France

**Keywords:** Sirtuin 1, endoplasmic reticulum stress, autophagy/mitophagy, cell death, cardioprotection

## Abstract

Many recent studies have demonstrated the involvement of endoplasmic reticulum (ER) stress in the development of cardiac diseases and have suggested that modulation of ER stress response could be cardioprotective. Previously, we demonstrated that the deacetylase Sirtuin 1 (SIRT1) attenuates ER stress response and promotes cardiomyocyte survival. Here, we investigated whether and how autophagy plays a role in SIRT1-afforded cardioprotection against ER stress. The results revealed that protective autophagy was initiated before cell death in response to tunicamycin (TN)-induced ER stress in cardiac cells. SIRT1 inhibition decreased ER stress-induced autophagy, whereas its activation enhanced autophagy. In response to TN- or isoproterenol-induced ER stress, mice deficient for SIRT1 exhibited suppressed autophagy along with exacerbated cardiac dysfunction. At the molecular level, we found that in response to ER stress (i) the extinction of eEF2 or its kinase eEF2K not only reduced autophagy but further activated cell death, (ii) inhibition of SIRT1 inhibited the phosphorylation of eEF2, (iii) eIF2α co-immunoprecipitated with eEF2K, and (iv) knockdown of eIF2α reduced the phosphorylation of eEF2. Our results indicate that in response to ER stress, SIRT1 activation promotes cardiomyocyte survival by enhancing autophagy at least through activation of the eEF2K/eEF2 pathway.

## 1. Introduction

The endoplasmic reticulum (ER) coordinates the synthesis, folding, and quality control of almost all secreted and membrane proteins. Perturbations that alter ER homeostasis cause the accumulation of unfolded/misfolded proteins in the ER lumen, leading to ER stress [1]. When ER stress occurs, the unfolded protein response (UPR) is triggered to restore normal ER function. The UPR is initiated by the activation of three proximal sensors, namely ATF6, IRE1, and PERK. Activation of these sensors leads to transcriptional and translational reprogramming to cope with the accumulation of unfolded proteins by reducing the translation of non-UPR proteins and upregulating the expression of UPR-related proteins such as ER chaperones, proteins that participate in ER-associated protein degradation (ERAD), and proteins of the autophagy pathway. Nevertheless, in the case of severe or chronic ER stress, if ER homeostasis fails to be reestablished, UPR assumes an adverse role and triggers the intrinsic pathway of apoptosis to eliminate damaged cells [2,3]. 

During the last decade, ER stress has emerged as an important mechanism involved in the development of various cardiac diseases, including ischemia, dilated cardiomyopathy, and heart failure [2,3]. Deletion of the ER stress response protein GRP78 or XBP1 has been shown to exacerbate the cardiac injury induces by ischemia, indicating that ER stress is cardioprotective in this context [4,5]. Besides, deficiency of the pro-apoptotic member of the UPR CHOP attenuates cardiac dysfunction induced by pressure overload [6], suggesting that ER stress participates in cardiac disease development. Therefore, these results lead to the notion that mild-to-moderate induction of ER stress triggers a restorative response that leads to cell adaptation, whereas severe or chronic ER stress by switching the protective signaling to the pro-apoptotic response contributes to the development of the cardiac disease. 

Autophagy, a major cellular pathway for clearance and recycling of damaged proteins or organelles such as mitochondria (a.k.a. mitophagy), is essential to heart homeostasis and function, but when excessive can contribute to cardiovascular disorders [7]. A complex interplay between autophagy and apoptosis is known to determine cell fate in response to ER stress, but the molecular events involved are still poorly understood [8]. The calcium/calmodulin-dependent enzyme eEF2K (eukaryotic elongation factor-2 kinase) that slows down the rate of protein translation by phosphorylating and inhibiting the function of the eEF2 (eukaryotic elongation factor-2) has been proposed to control the switch between autophagy and apoptosis in cancer cells in response to Akt inhibition [9]. However, little is known on the role of the eEF2K/eEF2 pathway in cardiovascular health and diseases. 

Sirtuin-1 (SIRT1), an NAD+-dependent lysine deacetylase, plays a cardioprotective role in response to various cardiac stress [10]. Mechanistically, the cardioprotective effects of SIRT1 have been partially attributed to its ability to influence autophagy, at least by directly deacetylating components of the autophagy machinery, including ATG5, ATG7, and LC3 [11]. However, whether and how SIRT1 modulates ER stress-induced autophagy in the heart has not been elucidated yet. 

We have previously shown that SIRT1 protects cardiomyocytes against ER stress-induced apoptosis by regulating the activation of the PERK pathway of the UPR [12]. In the present study, we hypothesized that SIRT1 might exert its cardioprotective effects by improving autophagy induced by ER stress. The ability of SIRT1 to regulate autophagy/mitophagy and apoptosis was tested in response to ER stress by using in vitro (H9c2 cells and isolated adult rat ventricular myocytes) and in vivo (SIRT1 iKO mice) models. The involvement of the eEF2K/eEF2 pathway in SIRT1-mediated regulation of autophagy was also investigated.

## 2. Materials and Methods

### 2.1. Animals and Treatments

C57BL/6J mice carrying both the UBC-Cre-ERT2 and the SIRT1floxΔE4/floxΔE4 alleles (UBC-Cre-ERT2 × SIRT1floxΔE4/floxΔE4) called herein SIRT1 iKO mice were described previously [13]. Excision of the exon 4 of SIRT1 was induced by tamoxifen (Sigma-Aldrich, Lyon, France, T5648) administration at 2 months of age (25 mg/kg i.p. daily × 4 days). To study ER stress in vivo, 4 weeks after tamoxifen treatment, male mice (n = 12) were given an i.p. injection of tunicamycin (TN, T7765, Sigma-Aldrich) 2 mg/kg body weight or the equivalent volume of vehicle (PBS) and euthanized by cervical dislocation 16 h after injection for molecular studies. Echocardiographic parameters were assessed 48 h after injection. To study the role of SIRT1 in a myocardial injury model, male mice (n = 5) were given a subcutaneous injection of isoproterenol (ISO) 150 mg/kg body weight or the equivalent volume of vehicle (NaCl 0.9%) at t0 and t6 h and were euthanized after 6 h for molecular studies. Echocardiographic parameters were assessed 48 h after injections. All experiments were performed in conformity with the European Community guiding principles in the care and use of animals (Directive 2010/63/EU of the European Parliament). Authorizations to conduct animal experiments were obtained from the French Ministère de l’Enseignement Supérieur, de la Recherche et de l’Innovation (n° B9201901, 3 November 2015).

### 2.2. Echocardiography

Transthoracic echocardiography was performed using a 15 MHz transducer under 2.5% isoflurane gas anesthesia. Two-dimensional-guided (2D) M-mode echocardiography was used to determine wall thickness and left ventricular chamber volume at systole and diastole and contractile parameters, such as fractional shortening (FS) and ejection fraction (EF).

### 2.3. Cell Culture and Reagents

The H9c2 rat cardiomyoblast cell line was purchased from ATCC (n° CRL-1446). Cells were cultured in DMEM medium supplemented with 100 U/mL penicillin, 100 mg/mL streptomycin, and 10% FBS (ThermoFisher Scientific, Les Ulis, France) at 37 °C under 5% CO2/95% air. EX527 was from Tocris (49843-98-3), STAC-3, and Endothall were from Santa Cruz (sc-222315 and sc-201325), A484954 was from Merck Chemicals (324516). Chloroquine and 3-Methyladednine were purchased from Sigma-Aldrich (C6628 and M9281). Cyto-ID^®^ Autophagy detection kit was from Enzo Life Sciences (ENZ-51031). Pilot experiments revealed that after 48 h of incubation, the LD50 of tunicamycin (concentration causing 50% cell death) was 10 µg/mL. This dose was thus used throughout this work.

### 2.4. Cell death Assessment by Flow Cytometry 

The fluorescent probe fluorescein diacetate (FDA) was used to assess cell viability. After treatment, cells were incubated for 10 min at 37 °C with 0.2 µg/mL FDA and analyzed by flow cytometry. Fluorescence of cells was analyzed on an FC500 cytometer (Beckman Coulter, Roissy, France). 

### 2.5. siRNA Transfection 

SIRT1, eEF2K, eEF2 and eIF2α siRNAs were purchased from Santa Cruz (sc-108043, sc-39012, sc-43542 and sc-35273). H9c2 cells were plated overnight at 30 × 10^4^ cells in 24-well plate and were transfected with 16 pg of either non-target control siRNA or SIRT1-, eEF2K-, eEF2- or eIF2α-specific siRNA according to manufacturer’s instructions. Twenty-four hours after transfection, the medium was removed, and cells were treated or not with TN for 24 h.

### 2.6. RNA Isolation and Quantitative RT-PCR

RNA was isolated from cultured cells using Zymo Research Quick RNA MiniPrep (R1054) according to the manufacturer’s instructions. Total RNA (1 μg) was reverse transcribed using BioRad iScript reverse transcription kit. Real-time PCR was performed using the SYBR^®^ green method on the CFX96 Touch™ Real-Time PCR Detection system (Bio-Rad, Marnes-la-Coquette, France) from 2.5 ng cDNA. mRNA levels for all target genes were normalized to Ywhaz and Polr2a. The PCR primers were obtained from Eurofins and are listed in Table S1. The results were quantified according to the Cq value method, where Cq is defined as the quantification cycle of PCR at which the amplified product is detected. The ratio (1+Etargetgene)^−(Cqsample−Cqcontrol)targetgene/(1+Ereferencegene)^−(Cqsample−Cqcontrol) gene was calculated, where E represented the efficiency of the quantitative PCR reaction. With this calculation, the expression of control was equivalent to one.

### 2.7. Adult Cardiomyocytes Isolation

Retrograde heart perfusion, according to the Langendorff method, was performed to isolate adult rat ventricle cardiomyocytes (ARVM) as previously described [14]. Cells were plated into dishes coated with laminin and were kept at 37 °C under 5% CO_2_/95% air for 24 h. Cells were then treated with 10 μg/mL TN with or without EX527 (20 μM), STAC-3 (1 μM), 3-MA (5 mM), or CQ (50 µM) at 37 °C under 5% CO_2_/95% air.

### 2.8. Evaluation of Adult Cardiomyocyte Death by Fluorescence Microscopy

To analyze cell death, adult rat ventricular myocytes (ARVM) were stained with 0.2 μg/mL of FDA (Sigma-Aldrich, F7378) for 10 min. FDA-negative cardiomyocytes were considered as dead cells. More than 1000 cells were counted for each condition, and representative micrographs were taken on a Leica fluorescence microscope (Leica Microsystems, Nanterre, France).

### 2.9. Electron Microscopy

H9c2 cells were fixed with 2% glutaraldehyde/0.2 M sodium cacodylate for 1 h at room temperature, pelleted and rinsed with 0.2 M sodium cacodylate/0.4 M sucrose for 1 h. Samples were epon-embedded, sliced, mounted on slides, and the images were obtained at the electron microscopy facility of INRA (Jouy-en-Josas, France). Left ventricular papillary muscles were isolated from 3 control and 3 TN-treated mouse hearts, fixated with 2% glutaraldehyde in cacodylate buffer (mM: 150 Na-Cacodylate, 2 CaCl_2_, pH 7.3) for 1 h, postfixated by 1% osmium tetroxide in cacodylate buffer for 30 min and stained with 1% aqueous solution of uranyl acetate. After dehydration in graded ethanol series and acetone, the tissue was embedded in Durcupan (Sigma-Aldrich, 44610-1EA). Ultrathin (58–60 nm) longitudinal sections were cut using an ultramicrotome (Power-Tome MTXL, RMC/Sorvall). The sections were mounted on formvar-coated copper grids, contrasted with lead citrate, and examined with a JEM 1200 electron microscope (Jeol) at 80 kV. 

### 2.10. Western Blot Analysis

Cells or tissues were lysed in RIPA lysis buffer (50 mM Tris-HCl pH 8, 150 mM NaCl, 1% Triton, 1 mM EDTA, 0.1% SDS, 0.5% deoxycholic acid) plus a cocktail of protease and phosphatase inhibitors (Roche, 11697498001 and 04 906 845 001), PMSF and a cocktail of deacetylase inhibitors (Sigma-Aldrich, 10837091001 and Santa Cruz sc-362323) for 30 min at 4 °C. Proteins (25 μg) were separated by 4%–20% Tris-Glycine or 4%–12% Bis-Tris gel electrophoresis and transferred to PVDF membranes (Fisher Scientific). Membranes were incubated overnight at 4 °C with the following antibodies: Anti-GRP94, anti-GRP78, anti-phospho-eEF2, anti-eIF2α and VDAC (Voltage-dependent anion channel) from Cell Signaling (#2104, #3183, #2331S, #2103 and #4866), anti-Actin from Santa Cruz (sc-8432), anti-LDH, anti-SIRT1, anti-CS from Abcam (ab52488, ab110304, ab96600), anti-LC3, anti-Parkin from Sigma-Aldrich (L7543 and P6248), anti-p62 from Novus Biologicals (H00008878-M01), anti-eEF2 from ThermoFischer (pa5-19617), anti-eEF2K from Proteintech (13510-1-ap). Proteins were detected on an iBright FL1000 Imager (Invitrogen) by using the ECL method according to the manufacturer’s instructions (Fischer Scientific, Illkirch, France). To determine the working range of each antibody, different protein quantities, antibody concentrations, and time of exposure were tested. To calculate the relative density (RD), ImageJ software was used, and the intensity of each protein was normalized to Actin. The data obtained were then expressed as the ratio of the intensity of the protein in treated cells to that of the corresponding protein in untreated cells. The level of protein phosphorylation was expressed as the ratio of the phosphorylated protein versus the total protein.

### 2.11. Autophagy Assessment

**Figure 1:**
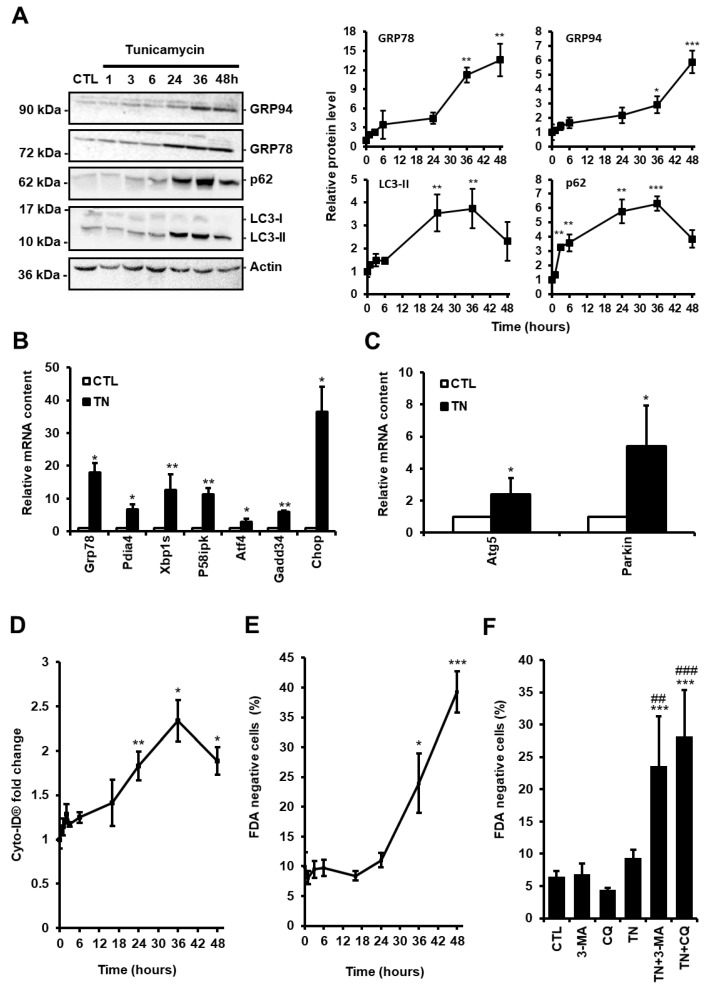
(**A**) H9c2 cells were treated with tunicamycin (TN, 10 µg/mL) for the indicated times and the levels of endoplasmic reticulum (ER) stress (GRP94 and GRP78) and autophagic (p62 and LC3-II) markers were analyzed by Western blot. To block autophagosome content degradation, 50 μM chloroquine (CQ) was added at the same time as tunicamycin (TN, 1 h) or 2 h before the end of tunicamycin treatment (TN, 3-48 h). Actin was used as a loading control. Relative protein levels are presented in graphs as mean ± S.E.M (n = 5). (**B**,**C**) Cells were left untreated or treated with tunicamycin (TN, 10 µg/mL) for 16 h and the relative mRNA content of UPR target genes (**B**) or autophagy genes (**C**) was quantified by RT-qPCR and expressed as fold change over untreated controls. Values represent mean ± S.E.M. (n = 5). (**D**) Autophagy and cell death were monitored by flow cytometry in H9c2 cells treated or not with tunicamycin (TN, 10 µg/mL) for the indicated times. Autophagy was detected after cell staining with Cyto-ID^®^. Mean fluorescence intensity ± S.E.M is presented (n = 5). (**E**) The percentage of cell death was assessed by Fluorescein Diacetate (FDA) assay. Results presented in the graph are expressed as the percentage of dead cells (FDA negative cells) (mean ± S.E.M., n = 5). (**F**) H9c2 cells were left untreated or treated with 10 μg/mL tunicamycin (TN) for 24 h ± 5 mM 3-Methyladenine (3-MA) or 50 μM chloroquine (CQ) pretreatment and cell viability was determined. Results presented in the bar graph are expressed as mean ± S.E.M. of percentages of dead cells (FDA negative cells, n = 5). * *P* < 0.05, ** *P* < 0.01, *** *P* < 0.005 versus control. ## *P* < 0.01, ### *P* < 0.005 versus TN. According to the results of the kinetics presented here, autophagy was studied at 24 h and cell death at 48 h in the rest of our study.

**Figure 2:**
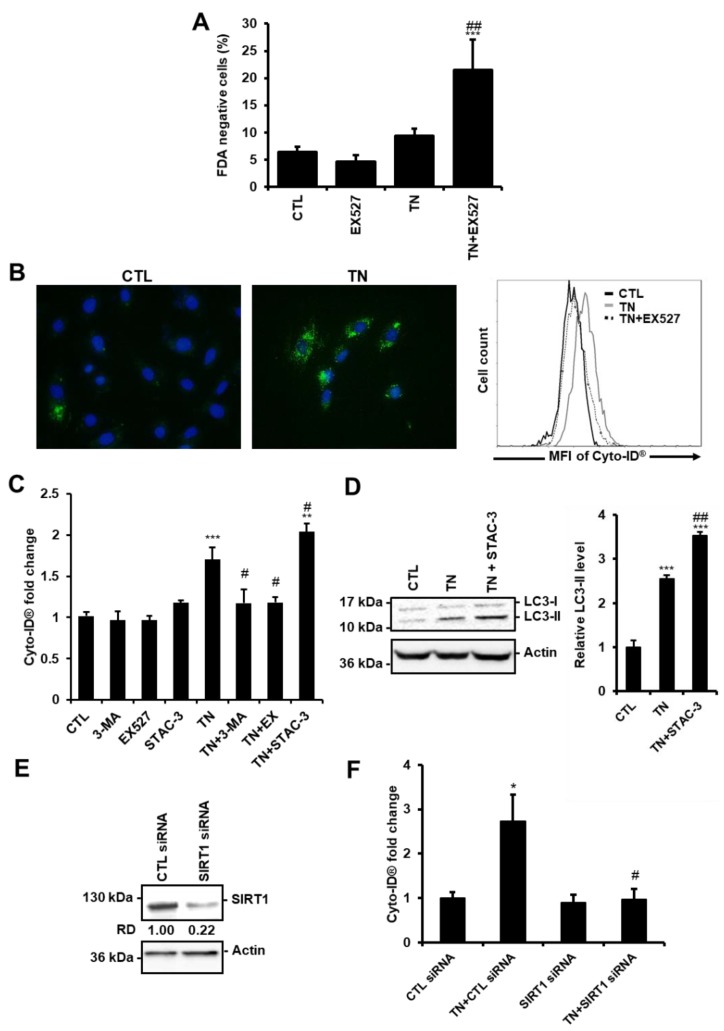
SIRT1 protects cardiac cells from ER stress by promoting autophagy. (**A**) H9c2 cells were left untreated or treated with 10 μg/mL tunicamycin (TN) for 24 h ± 50 μM EX527 (SIRT1 inhibitor) pretreatment and cell viability was assessed by Fluorescein Diacetate (FDA) assay. Results presented in the bar graph are expressed as the percentage of dead cells (FDA negative cells) (mean ± S.E.M., n = 5). (**B**,**C**) Cells were left untreated or treated for 24 h with 10 μg/mL tunicamycin (TN) ± 50 µM EX527, 5 mM 3-Methyladenine (3-MA) or 1 µM STAC-3 (SIRT1 activator) pretreatment and autophagy was monitored by flow cytometry after staining with Cyto-ID^®^ probe. (**B**) Typical staining of cells with Cyto-ID^®^ (green) and DAPI (blue) is shown. A representative flow cytometry overlay histogram showing the mean fluorescence intensity of Cyto-ID^®^ (MFI) in response to tunicamycin (TN) ± 50 µM EX527 is also presented. (**C**) Quantification of autophagy expressed as Cyto-ID^®^ fluorescence fold change ± S.E.M. (n = 7). (**D**) Cells were left untreated or treated with 10 μg/mL tunicamycin (TN) for 24 h ± 1 µM STAC-3 pretreatment and the levels of LC3-II were analyzed by Western blot. Actin was used as the loading control. Relative expression of proteins is presented in the bar graph as mean ± S.E.M (n = 7). (**E**) H9c2 cells were transfected with control or SIRT1 siRNA, and SIRT1 expression was assessed after 24 h by Western blot (n = 3). (**F**) H9c2 cells were transfected with control or SIRT1 siRNA for 24 h then treated for 24 h with tunicamycin (TN), and Cyto-ID^®^ fluorescence was measured by flow cytometry. Data in the bar graph represent mean ± S.E.M. (n = 3). * *P* < 0.05, *** *P* < 0.005 versus control. # *P* < 0.05, ## *P* < 0.01 versus TN.

Autophagy was assessed in H9c2 cells, ARVM, and mice hearts by Western blot analysis of LC3-II (and p62 in Figure 1). Chloroquine (50 µM) was added 2 h before the end of treatment (except for Figure 2) to inhibit autophagosome-lysosome fusion and thus to block autophagosome content and LC3-II degradation. Autophagy was also monitored in live cells using the Cyto-ID^®^ Autophagy Detection Kit. After treatment, cells were incubated with 0.1 µl of Cyto-ID^®^ reagent at 37 °C for 30 min, washed 3 times in a phenol-red-free culture medium containing 5% FBS, trypsinized and resuspended in 0.3 mL of culture medium for flow cytometry analysis. Cyto-ID^®^ fluorescence of cells was analyzed on an FC500 cytometer (Beckman Coulter).

### 2.12. Enzyme Activity 

Frozen tissue samples were weighed and homogenized (Bertin Precellys 24) in ice-cold buffer (50 mg/mL) containing HEPES 5 mM (pH 8.7), EGTA 1 mM, and 0.1% Triton X-100. Citrate Synthase activity was measured by detecting the increase in absorbance at 412 nm of 940 μL reaction buffer (200 mM Tris HCl, pH 8) containing 0.3 mM acetyl-CoA, 0.1 mM 5,5′-dithiobis-(2 nitrobenzoic acid) (DTNB), 0.5 mM oxaloacetic acid at 30 °C. A molar extinction coefficient of 13.6 L mol^−1^ cm^−1^ for DTNB was used. Cytochrome c oxidase activity was assayed from the decrease in absorbance at 550 nm caused by oxidation of ferrocytochrome c (reduced form) to ferricytochrome c (oxidized form) by Cytochrome c oxidase in 1 mL of phosphate buffer (50 mM K_2_HPO_4_, pH 7.4). The difference in extinction coefficients between reduced and oxidized cytochrome c was taken as 18.5 mM^−1^ cm^−1^ at 550 nm.

### 2.13. Preparation of Isolated Mitochondria

Mitochondria from H9c2 cells were isolated using standard procedures. Briefly, H9c2 cells were incubated for 10 min in 3 mL of IBc solution (in mM: 10 Tris-MOPS, 1 EGTA/Tris, 300 sucrose, 1%BSA, pH7.4) then minced with a tissue grind pestle. Homogenates were centrifuged at 2500 g for 10 min. The supernatant fraction was centrifuged at 10,000 g for 10 min. The pellet fraction was washed with 1 mL of IBc solution then centrifuged at 10,000 g for 10 min. The final pellet fraction was resuspended in 30 µL of RIPA buffer with anti-proteases and anti-deacetylases and analyzed by Western blot. To isolate cardiac mitochondria, hearts were minced in 4 mL of IB1 solution (in mM: 225 mannitol, 75 sucrose, 0.5% BSA, 0.5 EGTA, 30 Tris–HCl, pH 7.4) with a tissue grind pestle. Homogenate was centrifuged at 800 g for 5 min. The supernatant fraction was centrifuged at 9000 g for 10 min. The pellet fraction obtained was resuspended in 2 mL of IB2 buffer (in mM: 225 mannitol, 75 sucrose, 0.5% BSA, 30 Tris–HCl, pH 7.4) and centrifuged at 10000 g for 10 min. The pellet fraction was then resuspended in 2 mL of IB3 buffer (in mM: 225 mannitol, 75 sucrose, 30 Tris–HCl, pH 7.4) and centrifuged at 10000 g for 10 min. The final pellet fraction was resuspended in 50 µL of RIPA buffer with anti-proteases and anti-phosphates (1 mM PMSF, 1 mM Na_3_VO_4_, 2 µg/mL apoptinin, and 5 µg/mL leupeptin), 1% Triton™ X-100 and anti-deubiquitinase (2 mM N-ethylmaleimide, Sigma-Aldrich, 04259).

### 2.14. Native and Denaturing Immunoprecipitations

To analyze the level of eEF2 and eEF2K acetylation, cells were lysed in a denaturing IP buffer (1% SDS, 50 mM Tris-HCl, 5 mM EDTA, 10 mM DTT, 1 mM PMSF, 15 U/mL DNase, pH 7.4) supplemented with a protease and phosphatase inhibitors cocktail (Roche) and a cocktail of deacetylase inhibitors (Santa Cruz). Lysates were heated at 90 °C for 5 min and then diluted in a non-denaturing buffer to trap SDS (1% NP-40, 20 mM Tris-HCl, 2 mM EDTA, 137 mM NaCl, pH 8) supplemented with a cocktail of protease and deacetylase inhibitors. Lysine acetylated proteins, eEF2K and eEF2 proteins were immunoprecipitated using either anti-acetylated lysine or an anti-eEF2K antibody or anti-eEF2 antibody coated on G magnetic beads (Merck Millipore, Fontenay-sous-Bois, France, LSKMAGAG02). Mouse or rabbit anti-IgG was used as a control. To examine the physical interaction between SIRT1 or eIF2α and eEF2K or eEF2, cells were lysed in non-denaturing lysis buffer (20 mM Tris-HCl, 137 mM NaCl, 1% Triton X-100, 2 mM EDTA, pH 8) supplemented with a protease and phosphatase inhibitors cocktail (Roche) for 30 min at 4 °C. SIRT1, eIF2α, eEF2K, or eEF2 proteins were immunoprecipitated using anti-SIRT1, anti-eIF2α, anti-eEF2K, or anti-eEF2 antibodies coated on G magnetic beads (Merck Millipore, LSKMAGAG02). Mouse and rabbit anti-IgG were used as a control. Immunoprecipitated proteins were run on an SDS-PAGE and were revealed with anti-SIRT1, anti-eIF2α, anti-eEF2K, or anti-eEF2 antibodies.

### 2.15. Statistical Analysis

All data were presented as mean ± S.E.M. Data were analyzed using Sigma-Aldrich Stat (version 3.0, Systat Software, San Jose, CA, USA). One-way ANOVA was used to assess differences among groups followed by Newman–Keuls or Mann-Withney post hoc test. When the 2 groups were compared, differences were assessed by Student’s t-test. Differences between groups were considered significant if the P-value was * *P* < 0.05, ** *P* < 0.01, *** *P* < 0.005 versus control or WT mice. # *P* < 0.05, ## *P* < 0.01, ### *P* < 0.005 versus TN or SIRT1 iKO mice. 

## 3. Results

### 3.1. ER Stress Induces Protective Autophagy in Cardiac Cells

Autophagy is considered as a double-edged sword in the pathogenesis of cardiac diseases, acting in either protective or maladaptive ways, depending on the context [15]. To evaluate the role of autophagy in response to ER stress in cardiac cells, we treated H9c2 cells with the ER stressor tunicamycin (TN, 10 µg/mL) and measured the level of ER stress and autophagy markers over time. As expected, exposure to TN elicited ER stress, as demonstrated by the time-dependent upregulation of the ER chaperones GRP78 and GRP94 (Figure 1A). The expression of markers of ATF6 (Grp78, Pdia4, Chop), IRE1 (Xbp1s, P58ipk), and PERK (Atf4, Gadd34, Chop) pathways was increased, indicating that the three arms of the UPR were activated (Figure 1B). 

**Figure 3:**
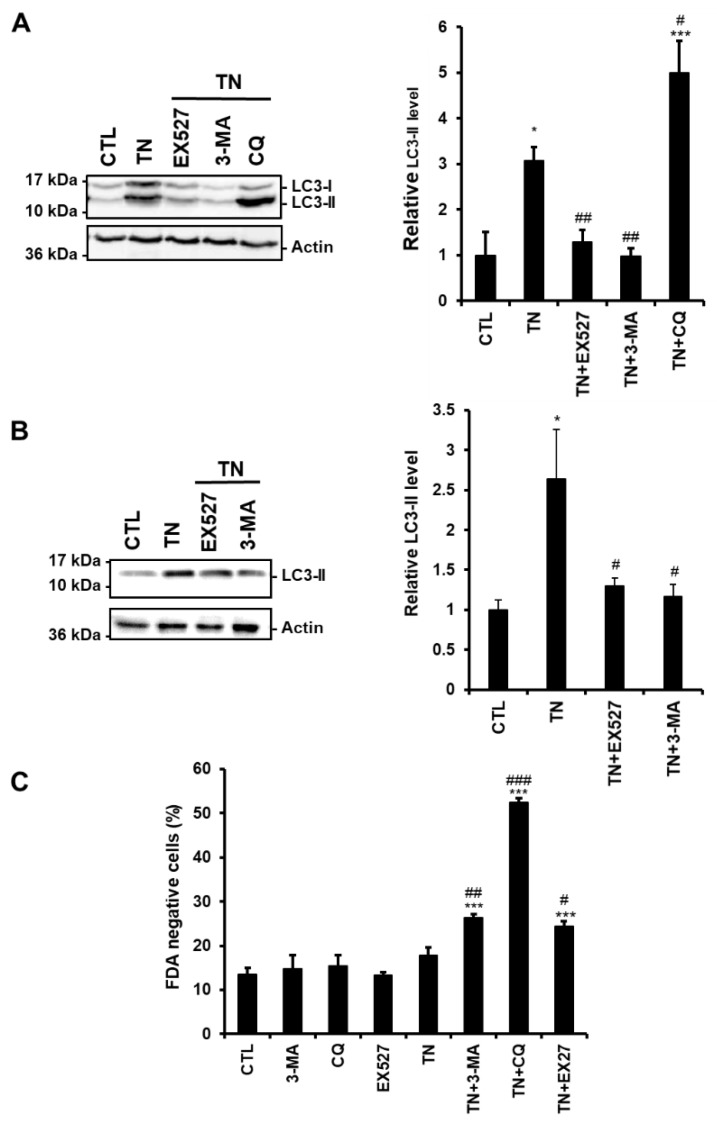
SIRT1 inhibition reduces the amount of LC3-II induced by ER stress. (**A**,**B**) The levels of the autophagic marker LC3-II were analyzed by Western blot in H9c2 cells (24 h) (**A**) or ARVM (4 h) (**B**) untreated or treated with tunicamycin (TN) (10 µg/mL) ± 50 µM EX527 (SIRT1 inhibitor), 5 mM 3-Methyladenine (3-MA) or 50 μM chloroquine (CQ) pretreatment. Actin was used as a loading control. Relative expression of proteins is presented in the bar graphs as mean ± S.E.M. (n = 4). (**C**) ARVM were left untreated or treated with 10 μg/mL tunicamycin (TN) for 24 h ± 50 µM EX527, 5 mM 3-Methyladenine (3-MA) or 50 μM chloroquine (CQ) pretreatment and cell viability was determined by Fluorescein Diacetate (FDA) assay. Results presented in the bar graph are expressed as mean ± S.E.M. of percentages of dead cells (FDA negative cells, n = 3). *P < 0.05, **P < 0.01, ***P < 0.005 *versus* control. #*P* < 0.05, ##*P* < 0.01, ###*P* < 0.005 *versus* TN.

The level of autophagy in response to ER stress was first evaluated by Western blotting of the most commonly used autophagy markers LC3-II and p62/SQSTM1. LC3-II is a phosphatidylethanolamine-conjugated form of LC3-I which is recruited to autophagosome membranes, and p62/SQSTM1 is an ubiquitin-binding protein which links LC3-II to ubiquitinated substrates. In the presence of autophagic flux inhibitors, such as chloroquine (CQ) or bafilomycin A, the level of LC3-II and p62/SQSTM1 have been shown to be proportional to the amount of autophagosomes [16]. H9c2 cells were treated for the indicated times with TN in the presence of CQ to inhibit the degradation of autophagosome content. In response to TN, autophagic flux blockade by CQ led to an initial increase in the amounts of p62/SQSTM1 and LC3-II that reached a maximum level at 36 h and then decreased (Figure 1A). Increased LC3-II levels can be associated with either enhanced autophagosome synthesis or reduced autophagosome turnover. Therefore, to assess the autophagic flux and discriminate between autophagy induction and inhibition of autophagosome degradation, the difference in the amount of LC3-II between TN-treated samples with and without CQ was analyzed (Figure S1A and Figure 3A). Blockade of autophagosome fusion with lysosome by CQ further increased the LC3-II protein level induced by TN, indicating that ER stress enhanced autophagic flux in H9c2 cells, as previously reported in atrial cardiomyocytes [17]. We also measured the expression level of the autophagy-related protein ATG5, which is indispensable for autophagic vesicle formation, and of the ubiquitin ligase Parkin, a well-described regulator of mitophagy, the selective subtype of autophagy, since they were previously reported to be transcriptionally up-regulated by the PERK/ATF4 pathway of the UPR [18,19]. As shown in Figure 1C, the expression of Atg5 and Parkin were upregulated upon ER stress in cardiac cells. In order to reinforce these results, H9c2 cells were stained with the Cyto-ID^®^ probe that allows flow cytometry analysis of autophagy in live cells by labeling autophagic compartments [20]. In parallel, cell death was assayed by FDA assay. Consistent with Western blot data, autophagy increased until 36 h post TN treatment and then decreased at 48 h (Figure 1D), whereas cell death was detectable only after 36 h (Figure 1E). Altogether, these results suggest that autophagy was initiated before cell death in response to ER stress. To investigate further the role of autophagy in the context of ER stress, H9c2 cells were treated with TN in the absence or the presence of the autophagy inhibitors CQ or 3-methyladenine (3-MA) for 24 h (Figure 1F) and 48 h (Figure S1B) and cell death was measured. Two different autophagy inhibitors were used to confirm that the effects observed were not due to the side effects of these molecules. After 24 h, while treatment with TN alone did not result in cell death, the percentage of dead cells was substantially increased in response to TN when autophagy was inhibited by CQ or 3-MA (Figure 1F). When the incubation time was extended to 48 h, these autophagy inhibitors further increased TN-induced cell death. Taken together, our results suggested that autophagy induced by ER stress was protective in cardiac cells.

### 3.2. SIRT1 Regulates ER Stress-Induced Autophagy in Cardiac Cells

Previously, we have reported that activation of the deacetylase SIRT1 protects the heart from ER stress-induced injury by decreasing apoptosis [12]. In agreement, cell death was increased in cardiac cells in response to ER stress when SIRT1 was pharmacologically inhibited by EX527, a selective and potent inhibitor of SIRT1 [21] (Figure 2A, 24 h, and Figure S1C, 48 h). We thus wondered whether the protective effect of SIRT1 involves the regulation of autophagy. The role of SIRT1 was examined in H9c2 cells by measuring Cyto-ID^®^ fluorescence and LC3-II levels. 

Figure 2B shows images obtained by fluorescence microscopy after Cyto-ID^®^ staining and representative results from flow cytometry analysis. As expected, in response to TN, inhibition of autophagy by 3-MA reduced Cyto-ID^®^ staining (Figure 2C). Inhibition of SIRT1 by EX527 decreased Cyto-ID^®^ fluorescence induced by TN to the same extent as did the autophagy inhibitor 3-MA. By contrast, activation of SIRT1 by STAC-3, a SIRT1-specific activator [13], further increased the fluorescence of Cyto-ID^®^ induced by ER stress. In agreement, the accumulation of LC3-II was more important in response to TN treatment when SIRT1 was activated by STAC-3 (Figure 2D). Moreover, in the context of ER stress, depletion of SIRT1 with siRNA (Figure 2E) decreased Cyto-ID^®^ fluorescence to basal level (Figure 2F), corroborating the results obtained with EX527. Together, these results showed that SIRT1 inhibition or knockdown decreased ER stress-induced autophagy, whereas activation of SIRT1 increased autophagy.

### 3.3. SIRT1 Inhibition Reduces the Accumulation of LC3-II Induced by ER Stress in Cardiac Cells

To determine more precisely the step of the ER stress-induced autophagic process affected by SIRT1, we compared the effects of the SIRT1 inhibitor EX527 to those of the autophagy inhibitors 3-MA and CQ on autophagy. Indeed, 3-MA blocks the initial step of autophagy (i.e., autophagosome formation and LC3-I lipidation) through inhibition of the PI3K, while CQ arrests the latter step of autophagy by interfering with the fusion of autophagosomes and lysosomes. H9c2 cells were treated with TN in the absence or the presence of EX527, 3-MA, or CQ, and the amount of LC3-II was analyzed by Western blot. 

Figure 3A shows that the accumulation of LC3-II induced by TN was further increased by CQ, whereas it was decreased by SIRT1 inhibition as observed when the initiation of autophagy was inhibited by 3-MA. To consolidate our results, the same experiments were conducted in adult rat ventricular myocytes (ARVM). As seen in H9c2 cells, EX527 and 3-MA reduced LC3-II accumulation induced by TN (Figure 3B). In addition, the modulation of TN-induced cell death by EX527 or autophagy inhibitors was also examined in ARVM (Figure 3C). After 4 h of treatment, TN did not affect cardiomyocyte viability. However, inhibition of autophagy (3-MA or CQ) or of SIRT1 (EX527) promoted cell death in response to TN, corroborating the protective roles of SIRT1 and autophagy observed in H9c2 cells (Figure 1 and Figure 2). Altogether, these data suggest that in the context of ER stress, SIRT1 inhibition interferes with autophagic flux upstream of LC3-II formation, rather than by blocking lysosomal degradation events. 

### 3.4. SIRT1 Deficiency Inhibits ER Stress-Induced Autophagy and Exacerbates Cardiac Injury 

**Figure 4:**
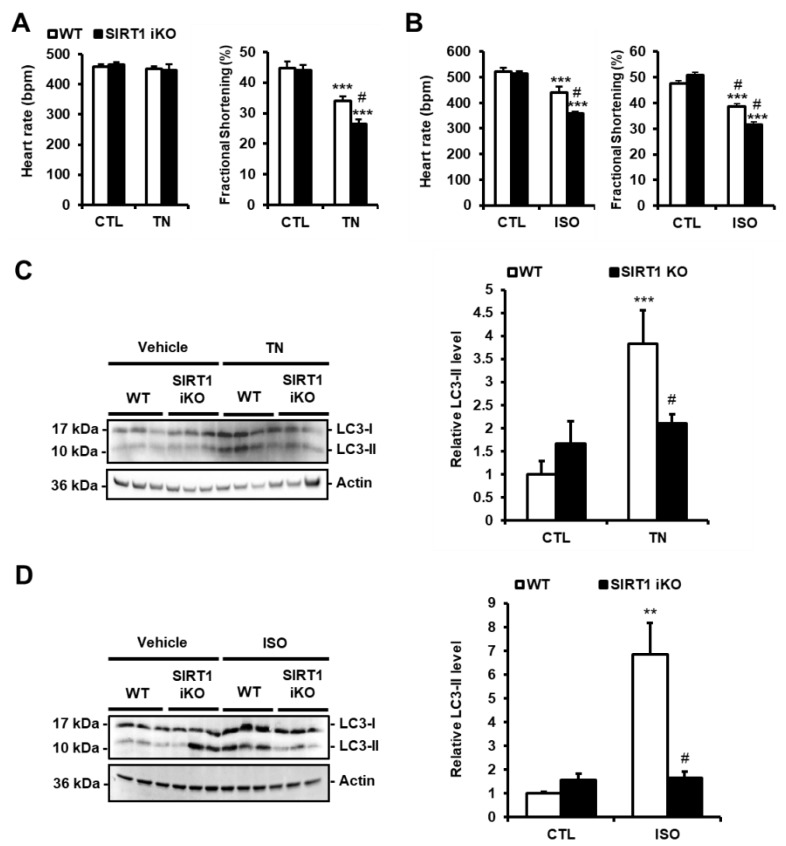
SIRT1 deficiency exacerbates cardiac dysfunction and decreases autophagy induced by ER stress. (**A**,**B**) WT and SIRT1 iKO mice were injected i.p. with tunicamycin (TN, 2 mg/kg) or vehicle (PBS) (**A**) or subcutaneously with Isoproterenol (ISO, 150 mg/kg) or vehicle (NaCl 0,9%) for 48 h (**B**) and heart rate and fractional shortening were recorded. Results are presented as mean ± S.E.M. (n = 12 for TN and n = 5 for ISO). (**C**,**D**) The levels of LC3-II were analyzed by Western blot in WT or SIRT1 iKO mice after TN (**C**) or ISO (**D**) treatment. Actin was used as a loading control. Relative expression of proteins is presented in bar graphs as mean ± S.E.M (n = 5–8). ** *P* < 0.01, *** *P* < 0.005 *versus* respective control. # *P* < 0.05, ## *P* < 0.01 *versus* WT TN or WT ISO.

To investigate the role of SIRT1 in the regulation of ER stress-induced autophagy in vivo, adult-inducible SIRT1 knockout (SIRT1 iKO) and control mice were injected with TN (2 mg/kg body weight). TN impaired cardiac function as shown by the decrease in fractional shortening (FS; Figure 4A) and ejection fraction (EF; Figure S2). Heart rate (Figure 4A), left ventricular internal diameters, or wall thickness (Figure S2) were not affected. SIRT1 deficiency significantly enhanced the diminution of FS and EF induced by TN. The role of SIRT1 was also examined in a model of cardiac injury. To this end, SIRT1 iKO and WT mice were subcutaneously injected with isoproterenol (ISO; 150 mg/kg b.w.), a β-adrenergic agonist. ISO elicits a catecholaminergic stress which has been well-documented to induce cardiac dysfunction associated with ER stress [12,22]. Following ISO challenge, heart rate, FS, and EF were decreased in WT mice, while left ventricular internal diameters and wall thickness were not affected (Figure 4B and Figure S3). The diminution of heart rate, FS, and EF induced by ISO was more important when SIRT1 was knocked out. Together, these data indicated that SIRT1 protects the heart from TN- and ISO-induced injury. To determine whether the cardioprotective effect of SIRT1 was mediated through the regulation of autophagy in vivo, the level of LC3-II was evaluated in WT and SIRT1 iKO mice after TN or ISO injection. The accumulation of LC3-II promoted by TN or ISO was returned to the basal level in SIRT1 iKO mice (Figure 4C,D), indicating that SIRT1 is required for autophagy induction in response to TN or ISO. These results corroborated those obtained in H9c2 cells and adult cardiomyocytes and suggest that SIRT1 protects the heart from ER stress-induced injury by promoting protective autophagy. 

### 3.5. ER stress-Induced Cardiac Mitophagy Is Regulated by SIRT1

**Figure 5:**
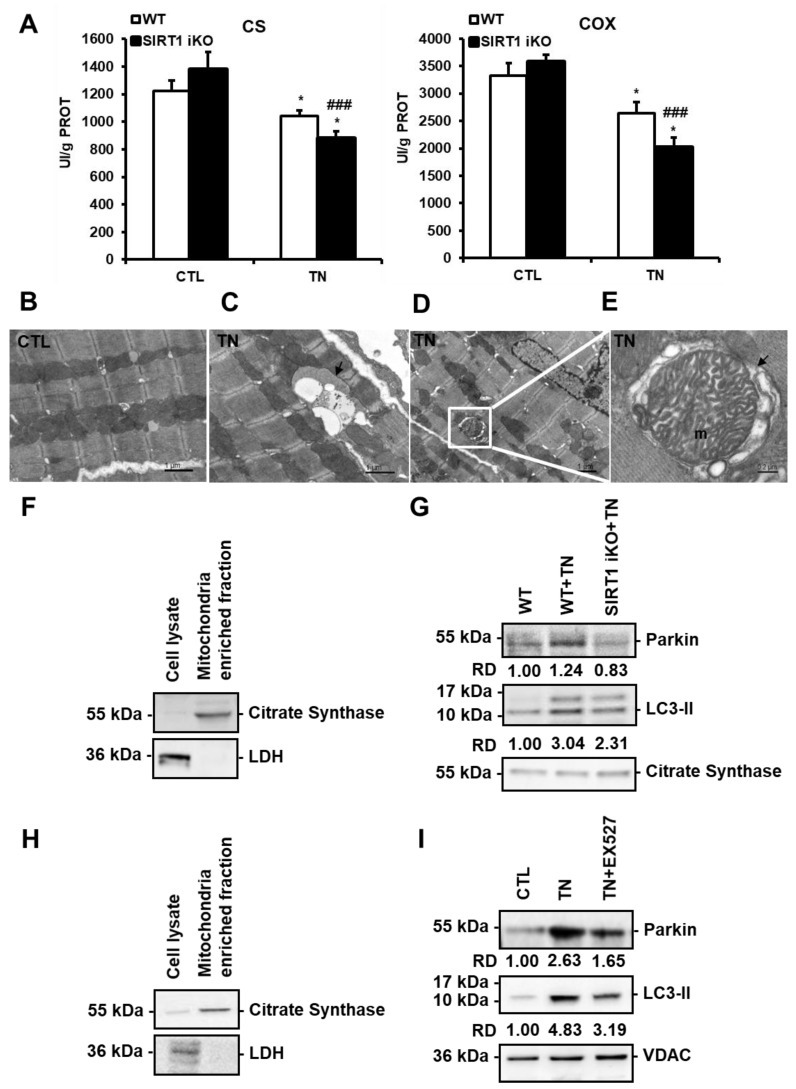
SIRT1 activates mitophagy in response to ER stress. (**A**) The activity of citrate synthase (CS) and cytochrome c oxidase (COX) in wild-type (WT) or SIRT1 iKO cardiac homogenates after vehicle or tunicamycin (TN) treatment. Enzymatic activities measured in UI/g proteins are presented in bar graphs as mean ± S.E.M. (n = 10). * *P* < 0.05 versus the respective control. ### *P* < 0.005 versus WT TN. (**B**–**E**) Electron micrographs of control (**B**) and tunicamycin (TN)-treated (**C**–**E**) cardiomyocytes showing autophagic/mitophagic vacuoles are presented. Figure 5C shows an autophagic vacuole (black arrow) surrounded by mitochondria. Figure 5D shows an autophagic vacuole containing a mitochondrion (white square). An enlarged micrograph of this vacuole is presented in Figure 5E. A swollen mitochondrion (m) can be observed in the autophagic vacuole (black arrow). (**F**,**G**) Immunoblots of mitochondrial fractions isolated from WT and SIRT1 iKO heart homogenates 16 h after tunicamycin (TN) injection (n = 5). (**F**) Purity of the mitochondrial fraction. (**G**) Western blot analysis of Parkin and LC3-II amounts in the mitochondrial fraction isolated from WT and SIRT1 iKO mouse hearts after administration of vehicle or tunicamycin (TN). Citrate synthase (CS) was used as a loading control. RD: Relative density. (**H**,**I**) Immunoblots of mitochondrial fractions isolated from H9c2 cells 16 h after tunicamycin (TN) treatment (n = 3). (**H**) Purity of the mitochondrial fraction. (**I**) Western blot analysis of Parkin and LC3-II amounts in the mitochondrial fraction of H9c2 cells treated or not with 10 μg/mL tunicamycin (TN) ± 50 µM EX527 (Sirt1 inhibitor). VDAC (Voltage-dependent anion channel) was used as a loading control. RD: Relative density.

Mitophagy, the selective removal of damaged mitochondria by autophagy, has been shown to be a crucial line of defense against myocardial stress [23]. We thus examined whether ER stress induced mitophagy in cardiomyocytes. First, the effects of ER stress and SIRT1 on mitochondrial function were analyzed by measuring the enzymatic activity of citrate synthase (CS) and cytochrome c oxidase (COX) in homogenates from WT or SIRT1 iKO mouse hearts. The activity of these two mitochondrial enzymes was decreased by TN (Figure 5A). This diminution was exacerbated by SIRT1 deficiency, indicating that SIRT1 reduced the impairment of the mitochondrial function caused by ER stress or increased the degradation of damaged mitochondria. To determine whether mitophagy was involved in ER stress-induced cardiac injury, ultrastructural analysis of cardiac tissues was carried out by transmission electron microscopy. The presence of double-membrane autophagic vacuoles containing cellular materials was observed in heart tissues from mice treated with TN (Figure 5C). In addition, some of these vacuoles contained damaged mitochondria, as shown in Figure 5D,E. The E3 ligase Parkin is known to translocate from the cytosol to damaged mitochondria to trigger mitophagy. To evaluate the translocation of Parkin to mitochondria in response to ER stress, subcellular fractionation experiments were performed. Mitochondria enriched fractions (Figure 5F) were prepared from hearts of WT, or SIRT1 iKO mice treated or not with TN, and the presence of Parkin and LC3-II was analyzed by Western blot (Figure 5G). In response to ER stress, the levels of mitochondrial Parkin and LC3-II were increased, showing an increase in mitophagy. The association of Parkin and LC3-II to mitochondria was reduced in the heart from SIRT1 iKO mice. These experiments were also conducted in H9c2 cells (Figure 5H,I and Figure S4). Transmission electron micrographs revealed autophagic vacuole formation following TN treatment (Figure S4). In addition, as observed in mice, the inhibition of SIRT1 by EX527 impaired the association of Parkin and LC3-II with mitochondria (Figure 5H,I). These results suggest that ER stress-induced mitophagy is regulated by SIRT1 in the heart.

### 3.6. SIRT1 Activates eEF2K/eEF2 Pathway to Regulate Autophagy and Cell Death in Response to ER Stress

**Figure 6:**
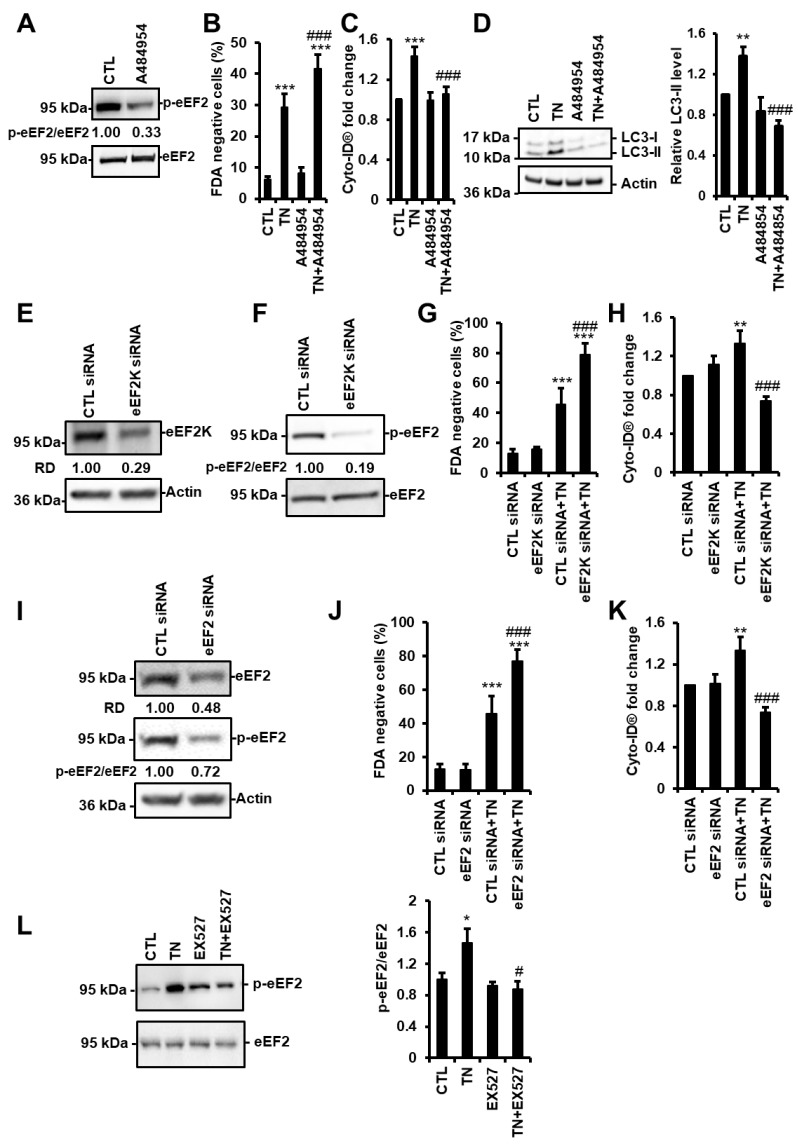
SIRT1 activates eEF2K/eEF2-dependent autophagy in response to ER stress. (**A**) H9c2 cells were treated with 10 µM A484954 for 4 h (eEF2K inhibitor) and the level of phospho-eEF2 (p-eEF2) was analyzed by Western blot (n = 7). The ratio of p-eEF2 vs. total eEF2 is presented. (**B**) H9c2 cells were left untreated or treated with 10 μg/mL tunicamycin (TN) for 48 h ± 10 µM A484954, and cell viability was determined by Fluorescein Diacetate (FDA) assay. Results presented in the bar graph are expressed as mean ± S.E.M. of percentages of dead cells (FDA negative cells, n = 11). (**C**) Autophagy was monitored by flow cytometry after Cyto-ID^®^ staining of H9c2 cells untreated or treated with 10 µg/mL tunicamycin (TN) for 24 h ± 10 µM A484954 (n = 11). (**D**) Cells were left untreated or treated with 10 μg/mL tunicamycin (TN) for 24 h ± 10 µM A484954 and the levels of LC3-II were analyzed by Western blot. Actin was used as a loading control. The relative mean expression level of LC3-II is presented in the bar graph. Error bars, S.E.M. (n = 5). (**E**,**F**,**I**) H9c2 cells were transfected with control, eEF2K or eEF2 siRNA, and eEF2K (**E**) or eEF2 (**I**) expression and eEF2 phosphorylation (**F**,**I**) were assessed after 24 h by Western blot. (**G**,**J**) H9c2 cells were transfected with control, eEF2K (**G**), or eEF2 (**J**) siRNA for 24 h, then treated for 48 h with tunicamycin (TN) and cell death (FDA negative cells) was measured by flow cytometry. Data in the bar graphs represent mean ± S.E.M. (n = 3). (**H**,**K**) H9c2 cells were transfected with control, eEF2K (**H**) or eEF2 (**K**) siRNA for 24 h, then treated for 24 h with tunicamycin (TN) and autophagy (MFI of Cyto-ID^®^) was measured by flow cytometry. Data in the bar graphs represent mean ± S.E.M. (n = 3). (**L**) phospho-eEF2 expression level was measured by Western blot in H9c2 cells after 4 h of 10 μg/mL tunicamycin (TN) ± 50µM EX527 (SIRT1 inhibitor) treatment. The ratio of p-eEF2 vs. total eEF2 is presented in the bar graph. Error bars, S.E.M. (n = 9). * *P* < 0.05, ** *P* < 0.01, *** *P* < 0.005 *versus* control. # *P* < 0.05, ### *P* < 0.005 *versus* TN.

The calcium/calmodulin-dependent kinase eEF2K phosphorylates and inhibits the eukaryotic elongation factor-2 (eEF2) under stressful conditions. In addition, the eEF2K/eEF2 pathway has been shown to control stress-induced autophagy and apoptosis in cancer cells [24]. As we demonstrated that SIRT1 regulates autophagy/mitophagy and cell death in cardiac cells in response to ER stress, we hypothesized that this regulation might involve the eEF2K/eEF2 pathway. To test this hypothesis, H9c2 cells were treated with A484954, a specific inhibitor of eEF2K, before TN treatment. The phosphorylation of eEF2 was reduced by about 70% in the presence of A484954 (Figure 6A), demonstrating that eEF2K was effectively inhibited in H9c2 cells. The inhibition of eEF2K by A484954 increased the percentage of dead cells induced by ER stress (Figure 6B). Conversely, this percentage was lowered by endothall, an inhibitor of the PP2A phosphatase that dephosphorylates eEF2 (Figure S5). A484954 also decreased the fluorescence of Cyto-ID^®^ and the accumulation of LC3-II triggered by TN (Figure 6C,D). When the expression of eEF2K was reduced by siRNA (Figure 6E), eEF2 phosphorylation was decreased (Figure 6F), TN-induced cell death was enhanced (Figure 6G), and autophagy was suppressed (Figure 6H). Similarly, silencing the expression and phosphorylation of eEF2 (Figure 6I) increased ER stress-induced cell death (Figure 6J) and decreased autophagy (Figure 6K). These results indicate that the eEF2K/eEF2 pathway promotes autophagy and inhibits cell death in cardiac cells in response to ER stress. The effect of SIRT1 inhibition on the level of phosphorylation of eEF2 was then examined. The phosphorylation of eEF2 promoted by TN was returned to basal level when SIRT1 was inhibited by EX527 (Figure 6L). Together, these results suggest that SIRT1 activates eEF2K/eEF2 pathway to promote cardioprotective autophagy in response to ER stress.

### 3.7. SIRT1-Mediated Activation of eEF2K/eEF2 Pathway Involves eIF2α

**Figure 7:**
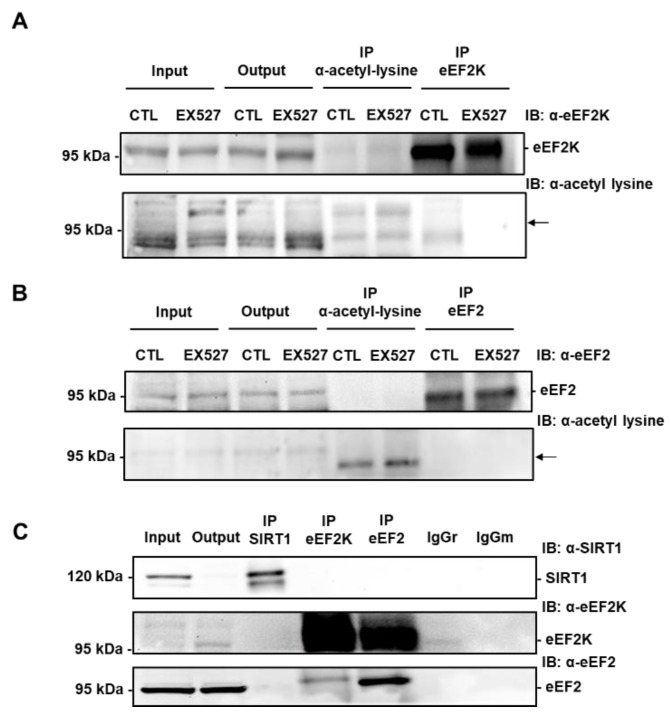
eEF2K and eEF2 are not acetylated on lysine and do not co-immunoprecipitate with SIRT1 in H9c2 cells. (**A**) Immunoprecipitation of lysine-acetylated proteins or eEF2K from lysates of H9c2 cells treated or not with 50 µM EX527 (SIRT1 inhibitor) followed by immunoblotting with anti-eEF2K or anti-acetyl lysine antibodies (n = 3). (**B**) eEF2 was immunoprecipitated from lysates of H9c2 cells treated or not with 50 µM EX527 and its level of acetylation was analyzed by immunoblotting with anti-acetyl lysine antibodies (n = 3). The location of eEF2K and eEF2 is shown by an arrow. (**C**) The physical interaction between endogenous SIRT1 and eEF2K or eEF2 was analyzed by co-immunoprecipitation. Either SIRT1 or eEF2K or eEF2 was immunoprecipitated from H9c2 lysates and the presence of SIRT1, eEF2K and eEF2 was analyzed by Western blot in each precipitate. Input: Input fraction; Output: Supernatant after immunoprecipitation; IP: Immunoprecipitate. Negative controls: IgGr: Rabbit IgG; IgGm: Mouse IgG (n = 3).

The observation that the deacetylase SIRT1 regulates eEF2 phosphorylation prompted us to assess whether eEF2K and/or eEF2 were acetylated on lysine and whether SIRT1 physically interacted with these proteins. To evaluate the acetylation of eEF2K and eEF2, proteins acetylated on lysine residues were pulled down from H9c2 cell lysates treated or not with EX527. Neither eEF2K nor eEF2 was detected in anti-acetyl lysine immunoprecipitates (Figure 7A,B). Immunoprecipitations of eEF2K or eEF2 followed by Western blotting with anti-acetyl lysine antibodies confirmed that eEF2K and eEF2 were not acetylated on lysine residues in H9c2 cells. The presence of eEF2K in eEF2 immunoprecipitates and vice-versa, indicates, as expected, that eEF2K and eEF2 physically interacted with each other. However, neither eEF2K nor eEF2 was found in SIRT1 immunoprecipitates (Figure 7C). The reverse experiment, immunoprecipitating eEF2K or eEF2 and immunoblotting with anti-SIRT1 antibodies, confirmed that SIRT1 was not associated with eEF2K or eEF2 in H9c2 cells. 

**Figure 8:**
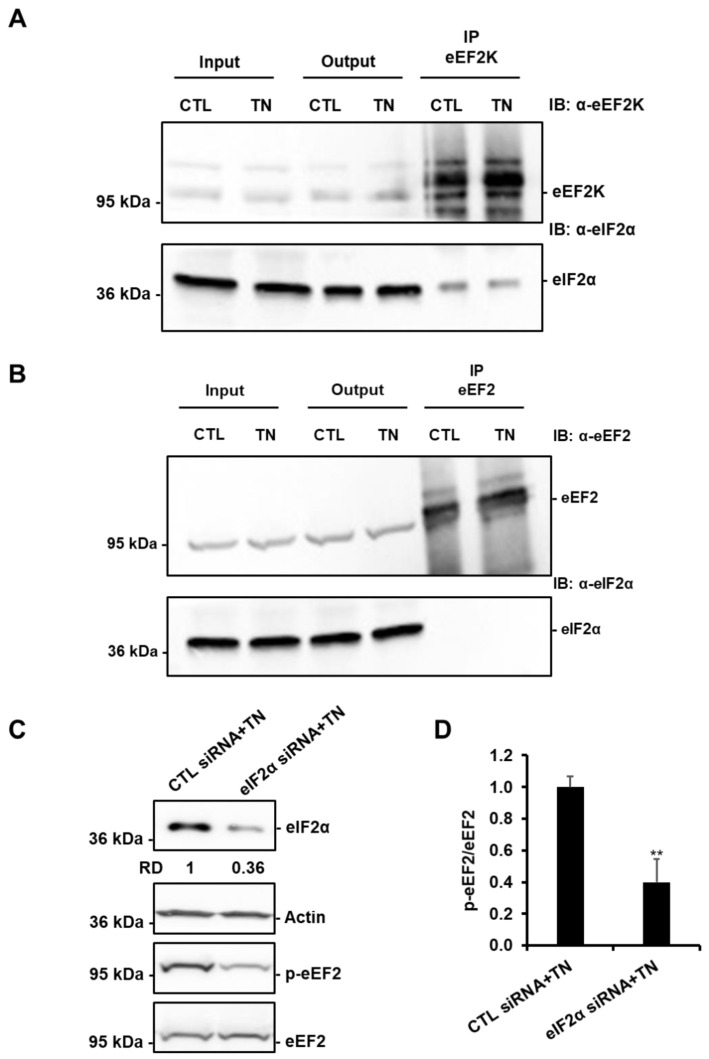
eIF2α is involved in the regulation of eEF2 phosphorylation in response to ER stress. eEF2K (**A**) and eEF2 (**B**) were immunoprecipitated from lysates of H9c2 cells treated or not with 10 μg/mL tunicamycin (TN), and the presence of eEF2K or eEF2 and eIF2α were analyzed by Western blot (n = 3). (**C**,**D**) H9c2 cells were transfected with control or eIF2α siRNA for 24 h, treated with tunicamycin (TN) for 1h30, and the level of eIF2α or phospho-eEF2 was analyzed by Western blot (RD: Relative density). The ratio of p-eEF2 vs. total eEF2 is presented in the bar graph. Error bars, S.E.M. (n = 4). ** *P* < 0.01 *versus* CTL siRNA+TN.

Since we had previously shown that SIRT1 protects the heart from ER stress through eIF2α deacetylation [12], we tested whether eIF2α was involved in SIRT1-mediated activation of eEF2K/eEF2 pathway. As shown in Figure 8A,B, eIF2α was co-immunoprecipitated with eEF2K, but not with eEF2. In addition, knockdown of eIF2α decreased the phosphorylation of eEF2 induced by TN (Figure 8C,D). These data suggest that activation of the eEF2K/eEF2 pathway by SIRT1 may involve eIF2α.

## 4. Discussion

Many recent studies have highlighted the importance of ER stress in the development and progression of cardiac disorders and have suggested that modulation of ER stress response could be cardioprotective [6,22,25]. Autophagy that is activated in response to multiple cellular stresses, including ER stress [8], is predominantly considered as a protective mechanism in the heart [26]. Moreover, we have previously demonstrated that SIRT1 protects cardiomyocytes against ER stress-induced apoptosis by modulating the activation of the PERK pathway of the UPR [12]. The main focus of this study was thus to investigate whether the cardioprotective effects of SIRT1 is related to modulation of ER stress-induced autophagy in cardiac cells and to investigate the mechanisms involved. We show that ER stress-induced autophagy is protective in cardiac cells. We also provide evidence that SIRT1 protects the heart by promoting autophagy through activation of the eEF2K/eEF2 pathway. 

Autophagy, as a major housekeeping process that allows recycling and clearance of damaged proteins and organelles, has been reported to play a beneficial or detrimental role in the myocardium depending on the context and the disease studied [15]. Under normal physiological conditions, basal levels of autophagy have been demonstrated to be essential for maintaining cardiac homeostasis because cardiomyocytes are terminally differentiated and cannot decrease their cellular waste by replication [27]. Under conditions of stress such as ischemia and β-adrenergic stimulation, autophagy mainly acts as a pro-survival mechanism by removing toxic protein aggregates and damaged organelles [28,29,30,31]. On the other hand, other studies recognized excessive autophagy as a detrimental mechanism in heart failure induced by pressure overload [32] or during reperfusion injury [33]. Here, we report that autophagy occurred before the onset of cell death in response to ER stress in cardiac cells. In addition, we found that inhibition of ER stress-induced autophagy by 3-MA or CQ triggers cell death, suggesting that activation of autophagy may counteract cell death and serve as a cardioprotective mechanism against ER stress. The impairment of the mitochondrial function associated with ER stress-induced heart injury ([34] and Figure 5A) led us to investigate the involvement of mitophagy, a form of autophagy that selectively eliminates damaged mitochondria. By electron microscopy and subcellular fractionation, we demonstrated that autophagic vacuoles can contain damaged mitochondria and that Parkin is translocated to mitochondria under ER stress. Parkin is an E3 ubiquitin-ligase that is recruited to damaged mitochondria to promote their degradation by the autophagy machinery through ubiquitination of mitochondrial substrates [35]. Therefore, our observations suggest that ER stress induces autophagy and selective removal of dysfunctional mitochondria by mitophagy in the heart. Several studies have reported that mitophagy deficiency exacerbates cardiac dysfunction, leading to the proposal that mitophagy plays an essential role in adapting to myocardial stress [31,36]. However, the role of mitophagy in response to ER stress in the heart is poorly characterized. On the basis of our findings, we propose that autophagy/mitophagy plays a protective role and is essential for the survival of cardiac cells undergoing ER stress. 

SIRT1 is a deacetylase that has been shown to protect cardiomyocytes against various cardiac stresses, at least by suppressing cell death or promoting autophagy [10,11,12,37,38]. Our previous work has shown that SIRT1 protects cardiomyocytes from ER stress-induced apoptosis by regulating the PERK arm of the UPR [12]. In the present study, we thus investigated whether SIRT1 can activate autophagic clearance to help to cope with ER stress and counteract cell death. We found that under ER stress when SIRT1 was inhibited, the percentage of dead cardiomyocytes was more important, whereas autophagy was reduced. In in vivo models of cardiac injury induced by TN or ISO, SIRT1 deficiency led to defective autophagy and aggravation of the cardiac dysfunction. In addition, the accumulation of LC3-II induced by ER stress was significantly decreased by SIRT1 inhibition, to the same extent as did 3-MA, which blocks the early stages of autophagy. These observations suggest that SIRT1 protects the heart from ER stress-induced cell death by enhancing autophagy.

It was shown that eEF2K controls the switch between autophagy and apoptosis in cancer cells [9]. Moreover, Py et al. identified the eEF2K/eEF2 pathway as an important effector of ER stress signaling in PC12 pheochromocytoma cells [39,40]. These results prompted us to examine the possibility that eEF2K/eEF2 pathway could play a role in the regulation of ER stress-induced autophagy by SIRT1. We showed (i) that ER stress induced an increase in the phosphorylation of eEF2, and (ii) that the knockdown of eEF2 decreased ER stress-induced autophagy while increasing cell death. In addition, the reduction of eEF2 phosphorylation by pharmacological inhibition or extinction of eEF2K led to the same outcome. These results indicate that the eEF2K/eEF2 pathway is involved in the regulation of the switch between autophagy and cell death in cardiac cells in response to ER stress. The observation that ER stress-induced phosphorylation of eEF2 is decreased when SIRT1 is inhibited suggests that SIRT1 is able to modulate the eEF2K/eEF2 pathway in our model of ER stress. Therefore, we propose that SIRT1 protects cardiomyocytes from ER stress by promoting autophagy through activation of the eEF2K/eEF2 pathway.

We next examined whether SIRT1 directly activated eEF2K and/or eEF2 through the regulation of their acetylation levels. We were not able to detect any acetylation of eEF2 or eEF2K on lysine in H9c2 cells. In addition, in co-immunoprecipitation assays, eEF2K was found to interact with eEF2, whereas no association between SIRT1 and eEF2K or eEF2 was detected. These results imply that SIRT1 does not directly activate eEF2K/eEF2 pathway in cardiac cells, suggesting that another factor regulated by SIRT1 is required for eEF2K/eEF2 pathway activation in response to ER stress. The PERK/eIF2α/ATF4 pathway of the UPR has been shown to regulate ER stress-induced activation of autophagy [8,41]. In addition, in cancer cells, ER stress-induced activation of ATF4 downstream of eIF2α phosphorylation was reported to promote autophagy by activating DDIT4, which in turn increases eEF2K activity through mTOR inhibition [42,43]. This suggests that in response to ER stress, PERK-mediated phosphorylation of eIF2α can regulate the activity of eEF2K and thus the phosphorylation state of eEF2 through ATF4. In a previous study, we have shown that SIRT1 specifically regulates the PERK pathway of the UPR through deacetylation of eIF2α on lysines K141/K143 [12]. Here, we found that eIF2α is co-immunoprecipitated with eEF2K and that depletion of eIF2α decreases ER stress-induced phosphorylation of eEF2. All the above observations suggest that in cardiac cells, SIRT1 could activate eEF2K/eEF2-dependent autophagy through regulation of the acetylation state of eIF2α. In the future, the availability of antibodies to acetyl-eIF2α (K141/K143) should provide a better understanding of the functional and structural mechanisms involved in the cross-talk between SIRT1/eIF2α and eEF2K/eEF2 pathways in the regulation of autophagy and cell death in cardiac cells. 

## 5. Conclusions

We demonstrated for the first time that SIRT1 activation protects cardiomyocytes against ER stress-induced cell death by enhancing autophagy at least through activation of the eEF2K/eEF2 pathway. Therefore, SIRT1 activation, by enhancing protective autophagy and reducing detrimental apoptosis, could constitute a promising therapeutic strategy to limit the development and progression of cardiac diseases associated with ER stress.

## Supplementary Materials

The following are available online at https://www.mdpi.com/2073-4409/9/2/426/s1, Figure S1: Effects of autophagy, and SIRT1 inhibitors on ER stress-induced autophagy and cell death. Figure S2: Echocardiographic parameters of WT and SIRT1 iKO mice in response to ER stress. Figure S3: Echocardiographic parameters of WT and SIRT1 iKO mice in response to isoproterenol (ISO). Figure S4: Analysis of ER stress-induced autophagy in H9c2 cells by electron microscopy. Figure S5: Effects of PP2A inhibition by endothall on ER stress-induced cell death. Table S1: Sequence of qPCR primers used in this study.
