# Supplementary material for: SIRT1 Protects the Heart from ER Stress-Induced Injury by Promoting eEF2K/eEF2-Dependent Autophagy
Source: Cells. 2020 Feb 12;9(2):426. doi: 10.3390/cells9020426 (PMC7072417; doi:10.3390/cells9020426)
Supplement: Supplementary file 1 [file cells-09-00426-s001.pdf]

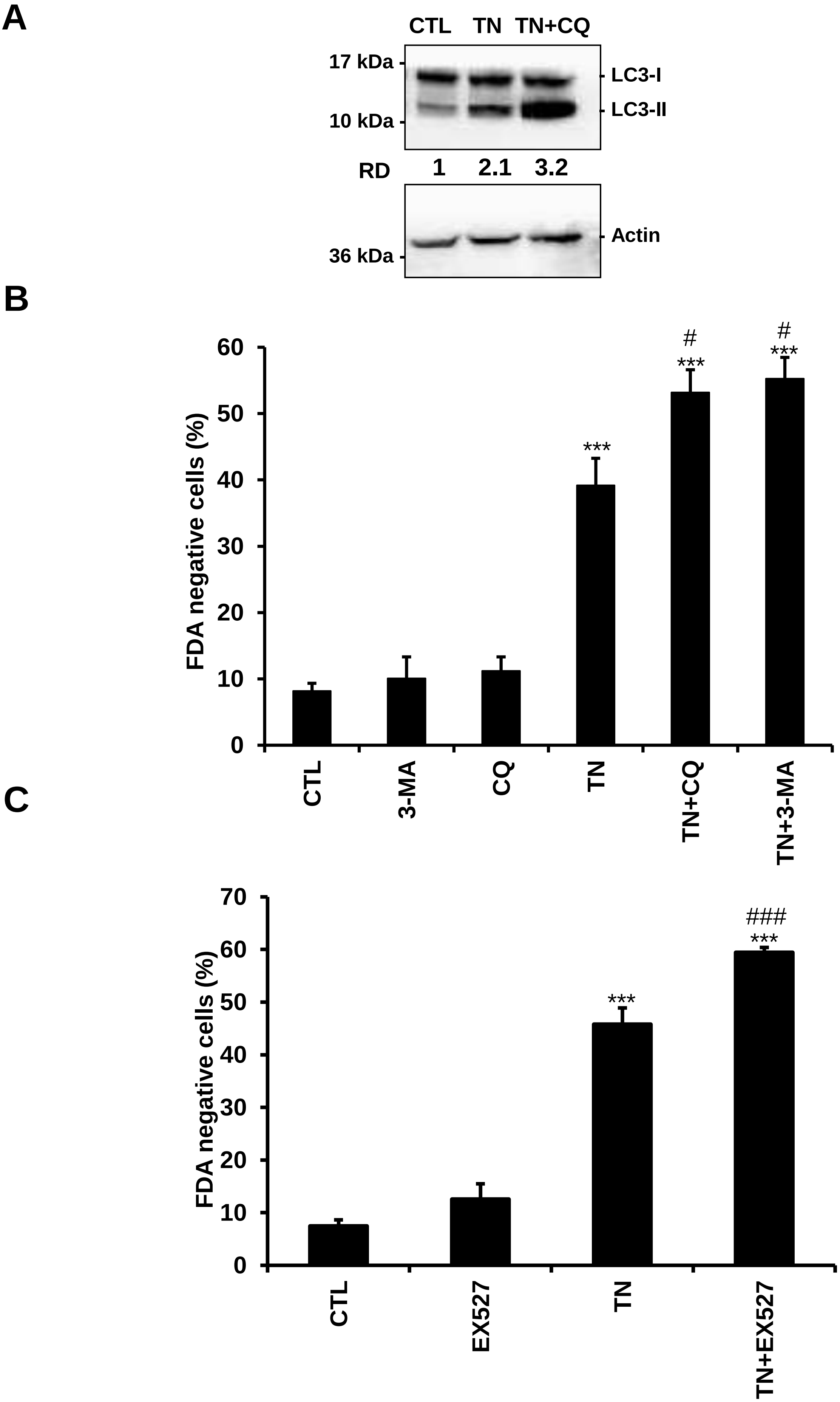

Pires Da Silva Figure S1

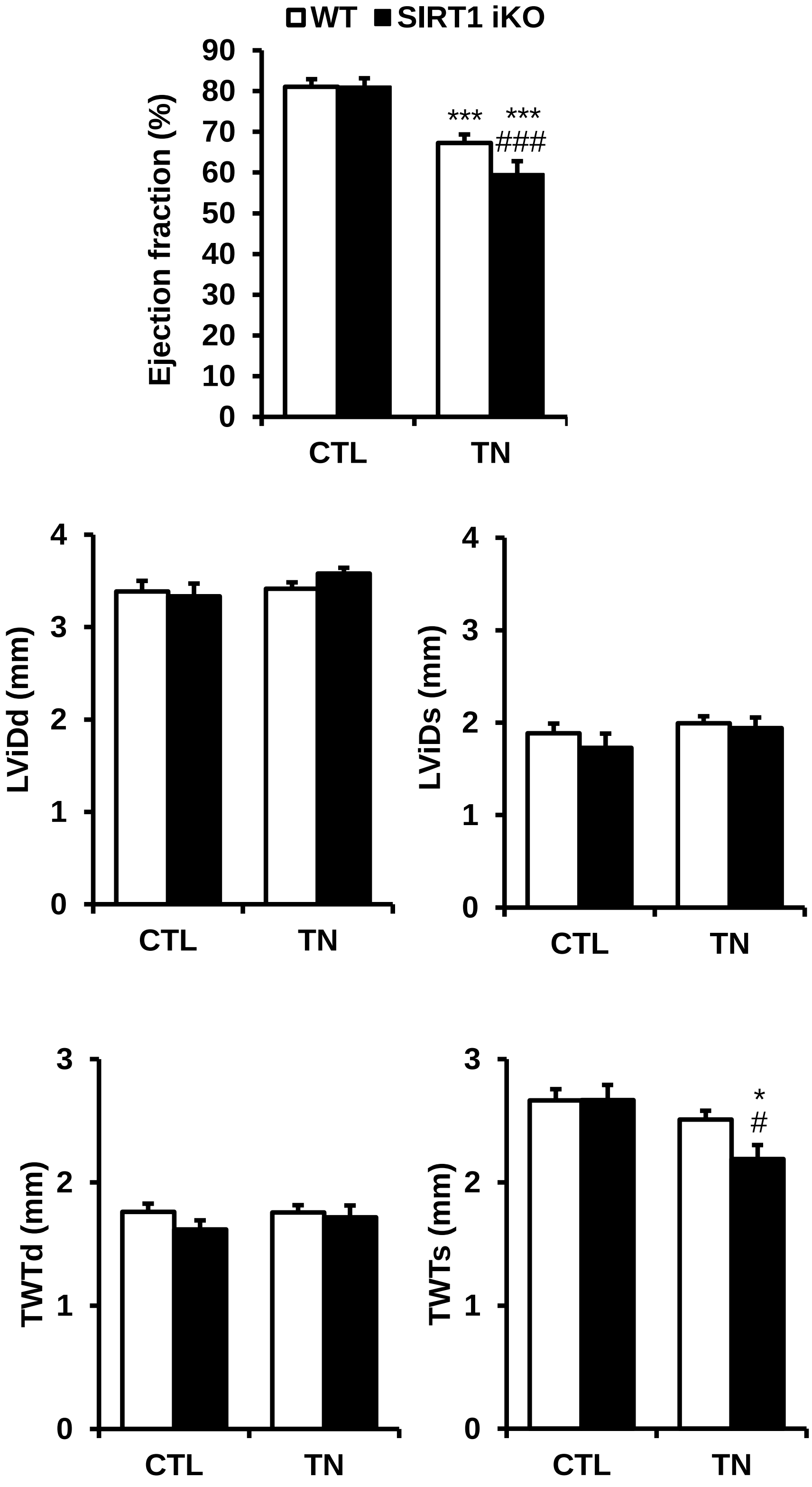

Pires Da Silva Figure S2

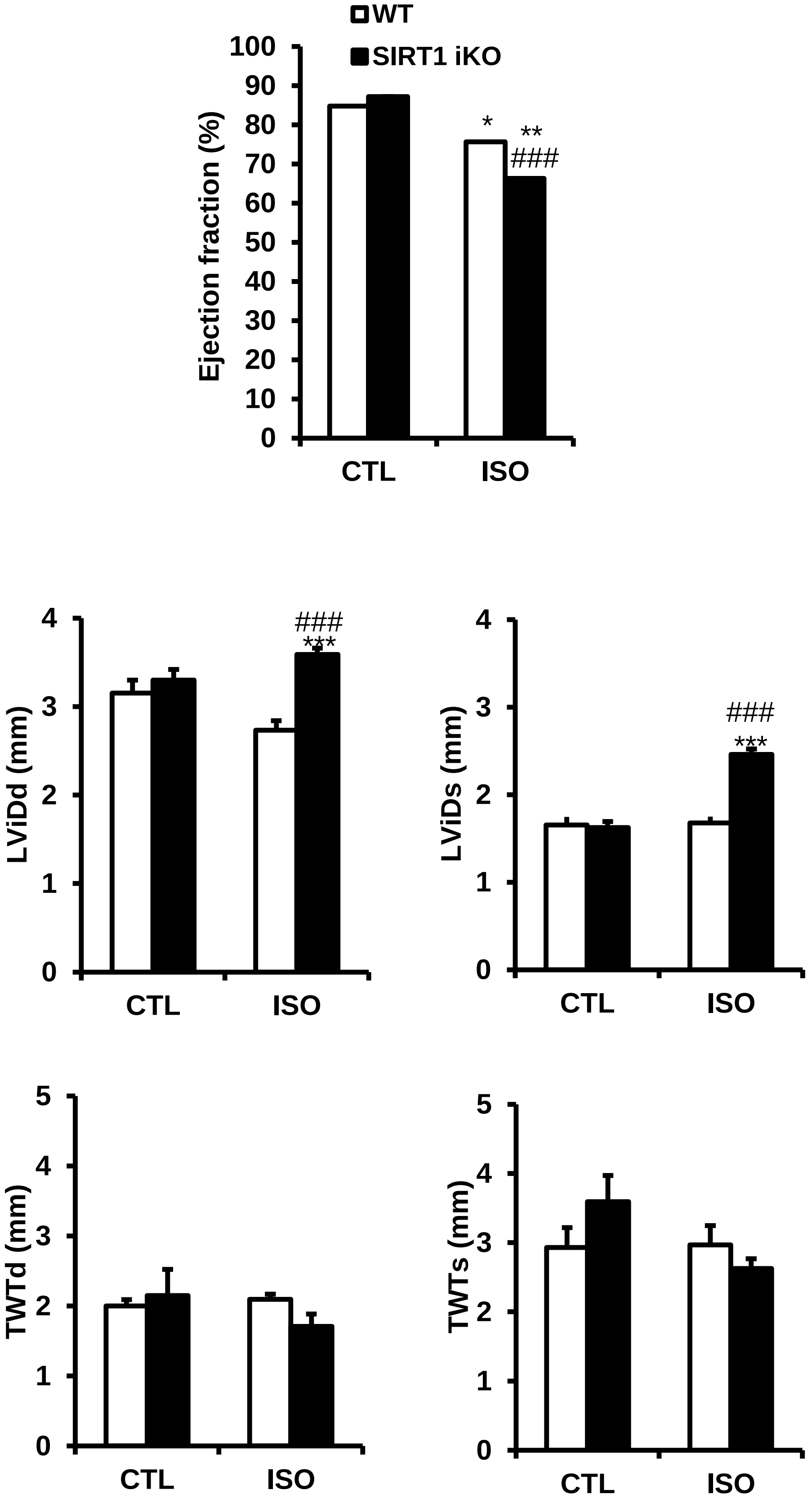

Pires Da Silva Figure S3

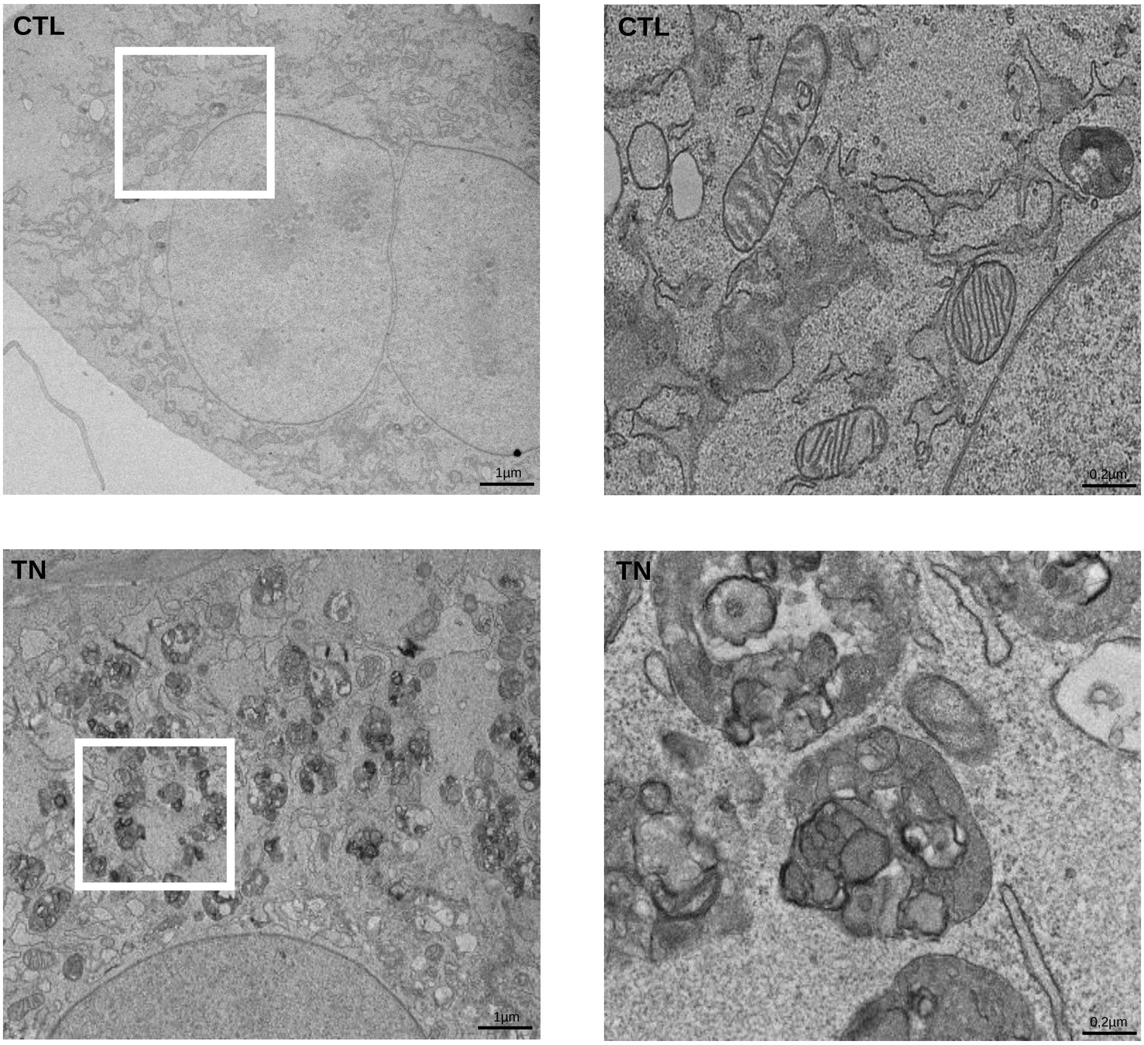

Pires Da Silva Figure S4

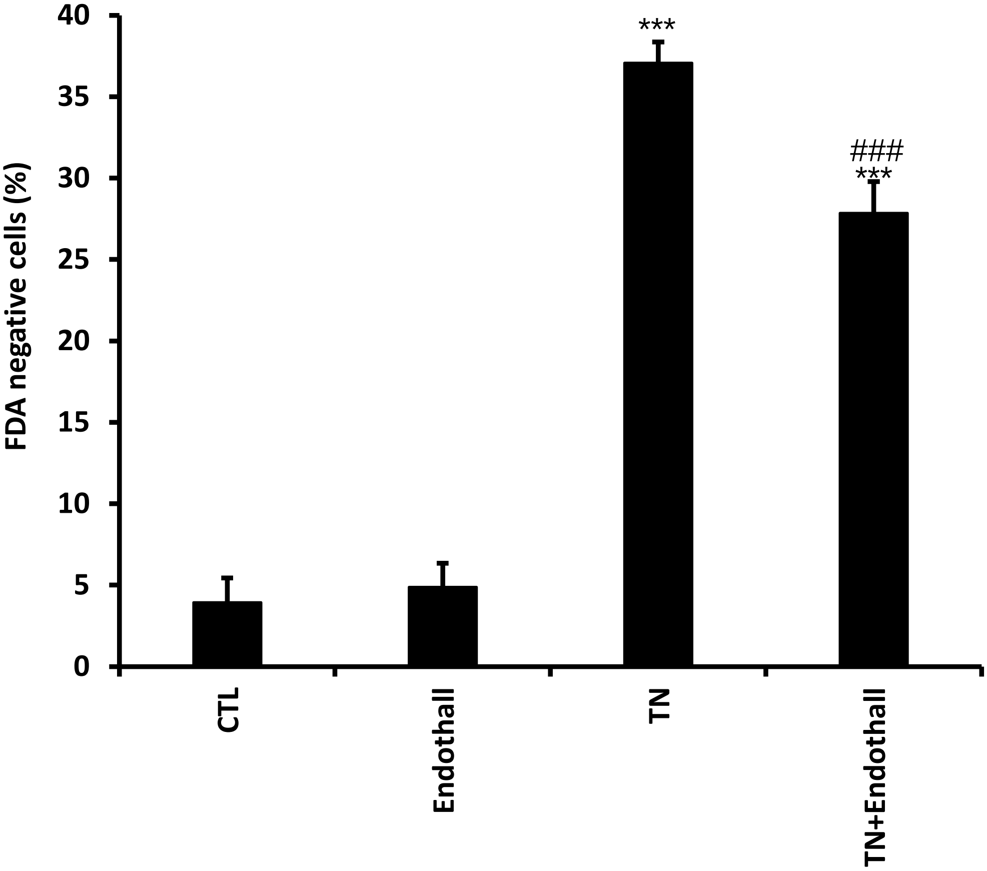

Pires Da Silva Figure S5

Table S1. Sequence of qPCR primers used in this study

Gene	Species	Forward primer	Reverse primer
**Atf4**	Rat mouse	AAACCTCATGGGTTCTCCAG	TCTCCAACATCCAACTGTCC
**Atg5**	Rat mouse	TTAGGGCAAGCTTTTTAGATGG	GCGGAAGGACAGACTTCTC
**Bip/Grp78**	Rat mouse	TGCAGCAGGACATCAAGTTC	TTTCTTCTGGGGCAAATGTC
**Chop**	Rat mouse	TATCTCATCCCCAGGAAACG	CAGGGTCAAGAGTAGTGAAGGTTT
**Gadd34**	Rat mouse	GGACCCTGAGATTCCTCTGA	GCCCAGACAGCAAGGAAAT
**P58ipk**	Rat mouse	CAGTTTCATGCTGCCGTAGA	GCTTTTGATTTGCCCATAGC
**Parkin**	Rat mouse	AACTCCAGCCATGGTTTCC	AAATCACACGCAACTGGTCA
**Pdia4**	Rat mouse	CTG ATT GGA CAC CTC CAC CT	AGG GGC AAG TTT CTT GCA G
**Xbp1s**	Rat mouse	TGCTGAGTCCGCAGCAGGTG	ACAGGGTCCAACTTGTCCAG

SUPPLEMENTAL FIGURE LEGENDS

Figure S1. Effects of autophagy and SIRT1 inhibitors on ER stress-induced autophagy and cell death.

(**A**) To assess the autophagic flux, the level of LC3-II was analyzed in response to TN with or without chloroquine. H9c2 cells were treated with TN (10 µg/ml) for 24h and the level of LC3-II was analyzed by western blot. To block autophagosome content degradation, 50 µM CQ was added 2h before the end of TN treatment. Actin was used as loading control. Relative density is indicated. (**B-C**) Cell viability of H9c2 cells after 48h TN treatment ± (**B**) 5 mM 3-MA or 50 µM CQ or (**C**) 50 µM EX527 pretreatment. Percentage of cell death (FDA negative cells) was assessed by flow cytometry. Results presented in graphs are expressed as mean ± S.E.M. of percentages of dead cells (FDA negative cells). ***P<0.005 *versus* control. #P<0.05, ###P<0.005 *versus* TN (n=5).

Figure S2. Echocardiographic parameters of WT and SIRT1 iKO mice in response to ER stress.

WT and SIRT1 iKO mice were injected i.p. with TN (2 mg/kg) or vehicle (PBS) for 72 h and transthoracic echocardiography was performed. LViDd: left ventricular internal dimension (diastole); LViDs: left ventricular internal dimension (systole); TWTd: total wall thickness (diastole); TWTs: total wall thickness (systole). Results are presented as mean ± S.E.M. ***P<0.005 *versus* respective control. #P<0.05, ###P<0.005 *versus* WT TN (n=12).

Figure S3. Echocardiographic parameters of WT and SIRT1 iKO mice in response to isoproterenol (ISO).

WT and SIRT1 iKO mice were injected subcutaneously with ISO (150 mg/kg) or vehicle (NaCl 0,9%) for 48 h and transthoracic echocardiography was performed. LViDd: left ventricular internal dimension (diastole); LViDs: left ventricular internal dimension (systole); TWTd: total wall thickness (diastole); TWTs: total wall thickness (systole). Results are presented as mean ± S.E.M. *P<0.05, **P<0.01, ***P<0.005 *versus* respective control. ###P<0.005 *versus* WT ISO (n=5).

Figure S4. Analysis of ER stress-induced autophagy in H9c2 cells by electron microscopy.

H9c2 cells were treated with TN (10 µg/mL) for 8 h, fixed and prepared as described in Materials and Methods and analyzed by electron microcopy.

Figure S5. Effects of PP2A inhibition by endothall on ER stress-induced cell death.

Percentage of cell death (FDA negative cells) was assessed by flow cytometry after TN (10 µg/mL) ± 10 µM Endothall treatment of cells for 48h. Results presented in graph are expressed as mean ± S.E.M. of percentages of dead cells (FDA negative cells, n=4). ***P<0.005 *versus* control. ###P<0.005 *versus* TN.

SUPPLEMENTAL TABLES

Table S1. Sequence of qPCR primers used in this study
